# Structures and comparison of endogenous 2-oxoglutarate and pyruvate dehydrogenase complexes from bovine kidney

**DOI:** 10.1038/s41421-022-00487-y

**Published:** 2022-11-22

**Authors:** Shiheng Liu, Xian Xia, James Zhen, Zihang Li, Z. Hong Zhou

**Affiliations:** 1 Department of Microbiology, Immunology, and Molecular Genetics, University of California, Los Angeles (UCLA), Los Angeles, CA USA; 2 California NanoSystems Institute, UCLA, Los Angeles, CA USA; 3 Molecular Biology Institute, UCLA, Los Angeles, CA USA

**Keywords:** Cryoelectron microscopy, Molecular biology

## Abstract

The α-keto acid dehydrogenase complex family catalyzes the essential oxidative decarboxylation of α-keto acids to yield acyl-CoA and NADH. Despite performing the same overarching reaction, members of the family have different component structures and structural organization between each other and across phylogenetic species. While native structures of α-keto acid dehydrogenase complexes from bacteria and fungi became available recently, the atomic structure and organization of their mammalian counterparts in native states remain unknown. Here, we report the cryo-electron microscopy structures of the endogenous cubic 2-oxoglutarate dehydrogenase complex (OGDC) and icosahedral pyruvate dehydrogenase complex (PDC) cores from bovine kidney determined at resolutions of 3.5 Å and 3.8 Å, respectively. The structures of multiple proteins were reconstructed from a single lysate sample, allowing direct structural comparison without the concerns of differences arising from sample preparation and structure determination. Although native and recombinant E2 core scaffold structures are similar, the native structures are decorated with their peripheral E1 and E3 subunits. Asymmetric sub-particle reconstructions support heterogeneity in the arrangements of these peripheral subunits. In addition, despite sharing a similar monomeric fold, OGDC and PDC E2 cores have distinct interdomain and intertrimer interactions, which suggests a means of modulating self-assembly to mitigate heterologous binding between mismatched E2 species. The lipoyl moiety lies near a mobile gatekeeper within the interdomain active site of OGDC E2 and PDC E2. Analysis of the twofold related intertrimer interface identified secondary structural differences and chemical interactions between icosahedral and cubic geometries of the core. Taken together, our study provides a direct structural comparison of OGDC and PDC from the same source and offers new insights into determinants of interdomain interactions and of architecture diversity among α-keto acid dehydrogenase complexes.

## Introduction

The pyruvate dehydrogenase complex (PDC; components suffixed by ‘p’) is an essential element of life that catalyzes the oxidative decarboxylation of pyruvate to acetyl-coenzyme A (acetyl-CoA). This key metabolic reaction links glycolysis to oxidative phosphorylation and the Krebs cycle. PDC is a member of the α-keto acid dehydrogenase complex family, alongside 2-oxoglutarate dehydrogenase complex (OGDC; ‘o’) and branched-chain α-keto acid dehydrogenase complex (BCKDC; ‘b’), which all perform analogous reactions in central metabolism^1,2^. Genetic studies have identified mutations in components of these multienzyme complexes linked to severe clinical consequences, including metabolic acidosis and neurodegeneration^3–7^. PDC also attracts interest in cancer biology for its role in modulating the Warburg effect to promote tumor anabolism^4,8–10^.

**Fig. 1:**
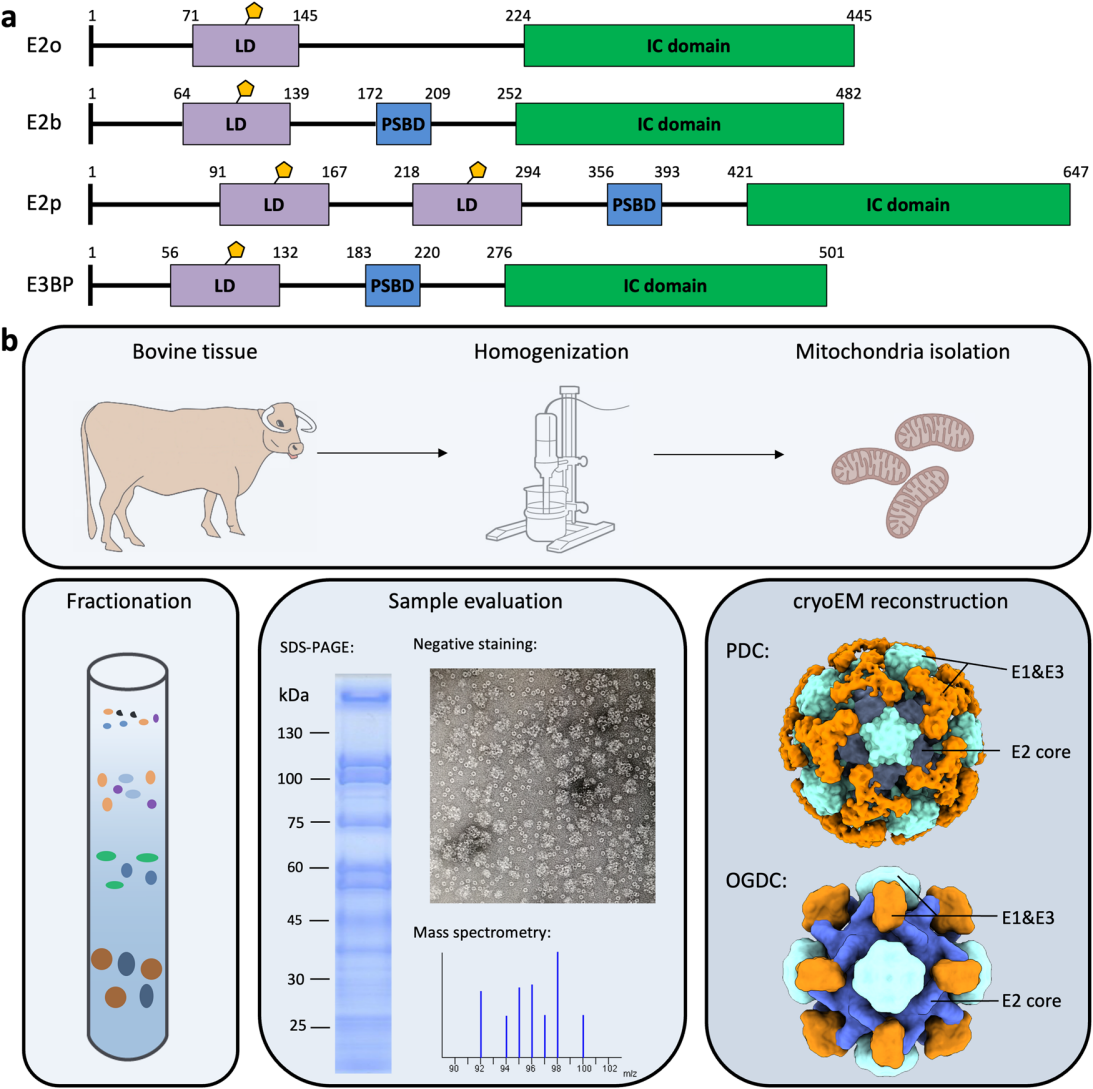
Preparation of bovine OGDC and PDC from native tissue lysate. **a** Organization of E2 and E3BP domains. **b** Workflow for extraction of PDC and OGDC from bovine tissue lysate. Bovine kidney was homogenized to isolate mitochondria. Mitochondrial lysates were then fractionated by sucrose gradient. Samples were evaluated for α-keto acid dehydrogenase complex by SDS-PAGE, negative stain, and mass spectrometry. Cryo-EM reconstruction of these samples yielded icosahedral and cubic cores surrounded by peripheral densities.

PDC, OGDC, and BCKDC are each comprised of multiple copies of a substrate-specific dihydrolipoamide acetyltransferase (E2p), dihydrolipoamide succinyltransferase (E2o), or dihydrolipoamide acyltransferase (E2b) inner catalytic (IC) core surrounded by multiple copies of the respective α-keto acid dehydrogenase (E1p, E1o, or E1b) and universal dihydrolipoamide dehydrogenase (E3) components^1,2^. Mammalian E2p or E2b is comprised of one or two lipoyl domains (LDs) followed by a peripheral subunit-binding domain (PSBD) and an IC domain that are connected by flexible linkers (Fig. 1a). Mammalian E2o has a similar composition but lacks the classical PSBD of E2p and E2b^11–13^. Each α-keto acid dehydrogenase complex proceeds through a three-step mechanism, starting with the decarboxylation of the α-keto acid substrate by E1 and transfer of an acetyl, acyl, or succinyl functional group to lipoate in the LD of E2. Then, the functionalized LD localizes to the IC core, where the functional group is transferred from the lipoyl moiety to CoA. Lastly, E3 reoxidizes the lipoyl moiety, which enables the process to repeat with the production of NADH. The LD translocates between these active sites through a flexible swinging arm mechanism^14^.

Although each E1 and E2 subunit in an α-keto acid dehydrogenase complex is highly similar in both sequence and overall structure to its counterpart in the other classes of the family and the same E3 protein is shared by all classes within a tissue^1,15,16^, there are notable differences in the architecture of how these components assemble. E1o is a homodimer, and E1p is either a homodimer or a heterotetramer in which each polypeptide of the homodimer is divided into an alpha and a beta subunit^17,18^. In the gram-negative bacteria *Escherichia coli* and *Azotobacter vinelandii*, PDC, OGDC, and BCKDC cores all have a 24-mer cubic architecture. However, in eukaryotes and gram-positive bacteria, PDC cores adopt a different 60-mer icosahedral architecture despite OGDC and BCKDC cores retaining a cubic geometry^16,18–24^. As an exception, actinobacteria distinctly lack assembled cores of E2p and also possess a unique OdhA protein that fuses the E2o catalytic domain to E1o^25^. Eukaryotic PDC has an additional noncatalytic E3-binding protein (E3BP) that specifically binds E3 to the core and is structurally similar to E2^16,19,26–32^. In mammals, E3BP substitutes for E2p in the core scaffold at possible stoichiometric ratios of 48:12 or 40:20 E2p:E3BP in an unknown arrangement^28,31^. Although peripheral E1 and E3 subunits are bound to the core by a discrete PSBD located in E2 or E3BP, eukaryotic E2o distinctly lacks a discrete PSBD for the binding of E1 and E3^11–13^. Instead, E3 binds an N-terminal region of E1o that shares antisera reactivity with E2p and E3BP and that is similar to them in sequence^13,33,34^. In turn, E1o binds to E2o at a region comprised of residues from the IC domain and the preceding linker^13,22^. Nonetheless, the subunits of each α-keto acid dehydrogenase complex self-assemble into their respective complexes^24^.

Much of our current structural understanding of α-keto acid dehydrogenase complexes is derived from crystal structures of recombinantly expressed proteins. However, the flexible assembly of intact α-keto acid dehydrogenase complexes is unamenable to crystallography, and components were often crystallized individually. Structures from these individual proteins are unable to fully capture how multiple components come together and function as a complex. In addition, crystallization may impose artificial order on protein side chains^35^. In the absence of the structure of native complexes, we have an insufficient understanding of how these subunits are organized into functional units. Recently, structures of native PDC from fungus and bacteria became available from cryo-electron microscopy (cryo-EM)^16,23,32,36,37^. In the intact fungus PDC structures, E3BP was observed as an additional component appended to the interior of the core instead of substituting for an E2p subunit of the core like in mammals and contains a conserved fold found in other E2 proteins^16,32,36^. From bacteria cultured in minimal media to minimize the activity of PDC, cryo-EM density of the lipoyl domains interacting with the PDC IC domains could be observed. Despite these exciting progresses, no such endogenous, intact α-keto acid dehydrogenase complexes have been determined for mammals.

In this study, we report the cryo-EM structures of endogenous OGDC and PDC E2 IC domain cores extracted from bovine kidney tissue at 3.5 Å and 3.8 Å resolution, respectively. We observe peripheral subunits around the core and the lipoyl moiety substrate within the E2 active site for both complexes. By comparing structures of PDC and OGDC from the same source, we identify distinct interactions in the IC domain trimer and in the twofold related intertrimer interface, which may direct self-assembly within a milieu of similar components.

## Results

### Extraction, cryo-EM analysis, and identification of native, intact OGDC and PDC

**Fig. 2:**
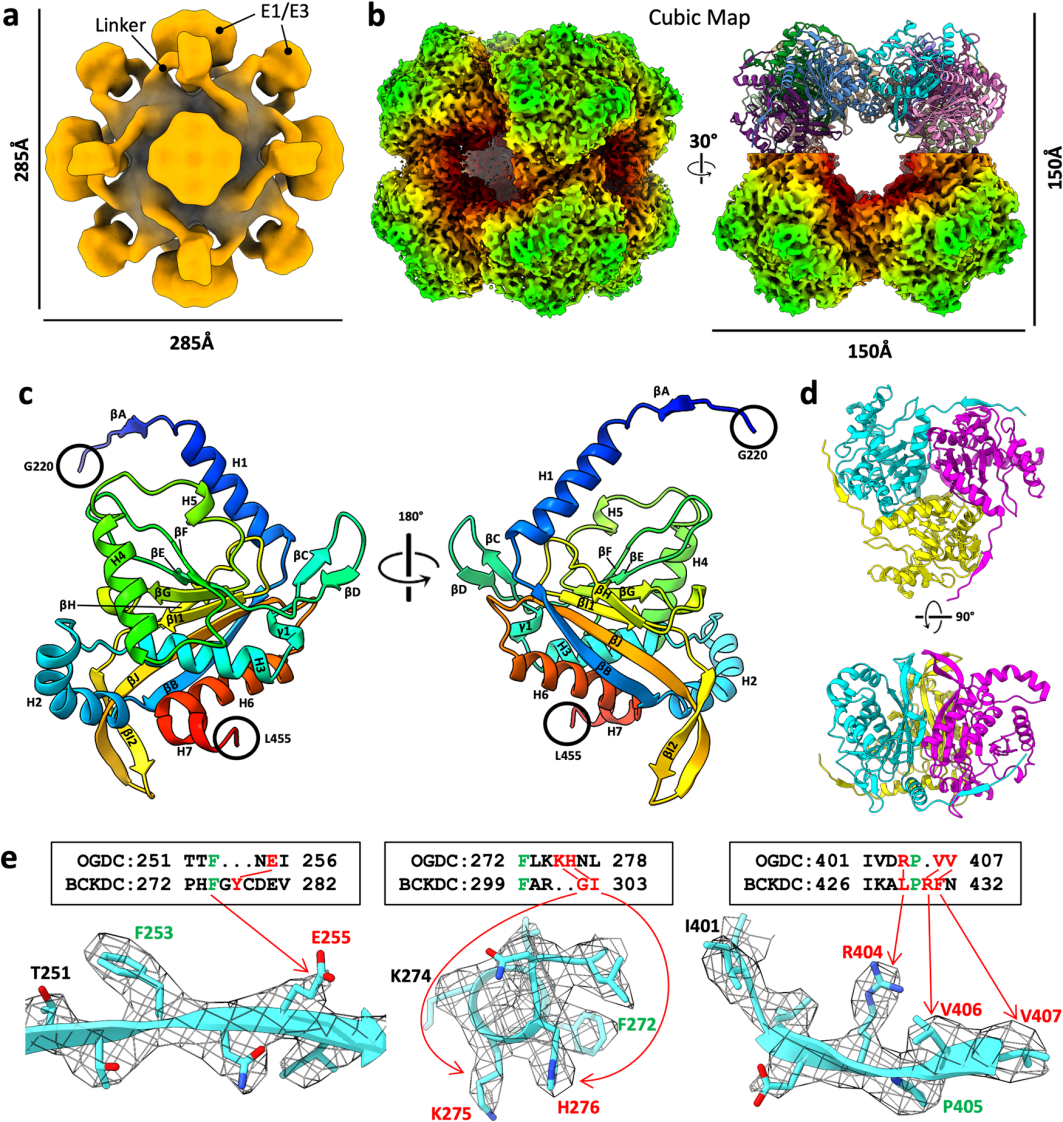
Cryo-EM reconstruction and identification of bovine OGDC core. **a** 3D reconstruction of the full OGDC with octahedral symmetry showing external densities (orange) around the core density (gray). Linkers extend from each E2o subunit toward a density along the edges of the core. Additional large densities are present atop the faces of the core. **b** Two views of the cryo-EM density map of E2o core colored by radius, with half of our E2o atomic model assembly in the right. **c** Rainbow-colored ribbon of E2o IC domain atomic model with secondary structures labeled. Resolvable N-terminal residues (blue) and C-terminus (red) are indicated. **d** Structure of the E2o IC domain trimer, colored by subunits. **e** Sequence alignment of E2o and E2b with side-chain fit of corresponding E2o sequence into cryo-EM density map. Green letters indicate conserved residues between E2o and E2b that establish landmark features in cryo-EM density. Red letters and arrows indicate mismatch of residue identity in E2b with corresponding cryo-EM density, thus establishing the map to be that of E2o.

**Fig. 3:**
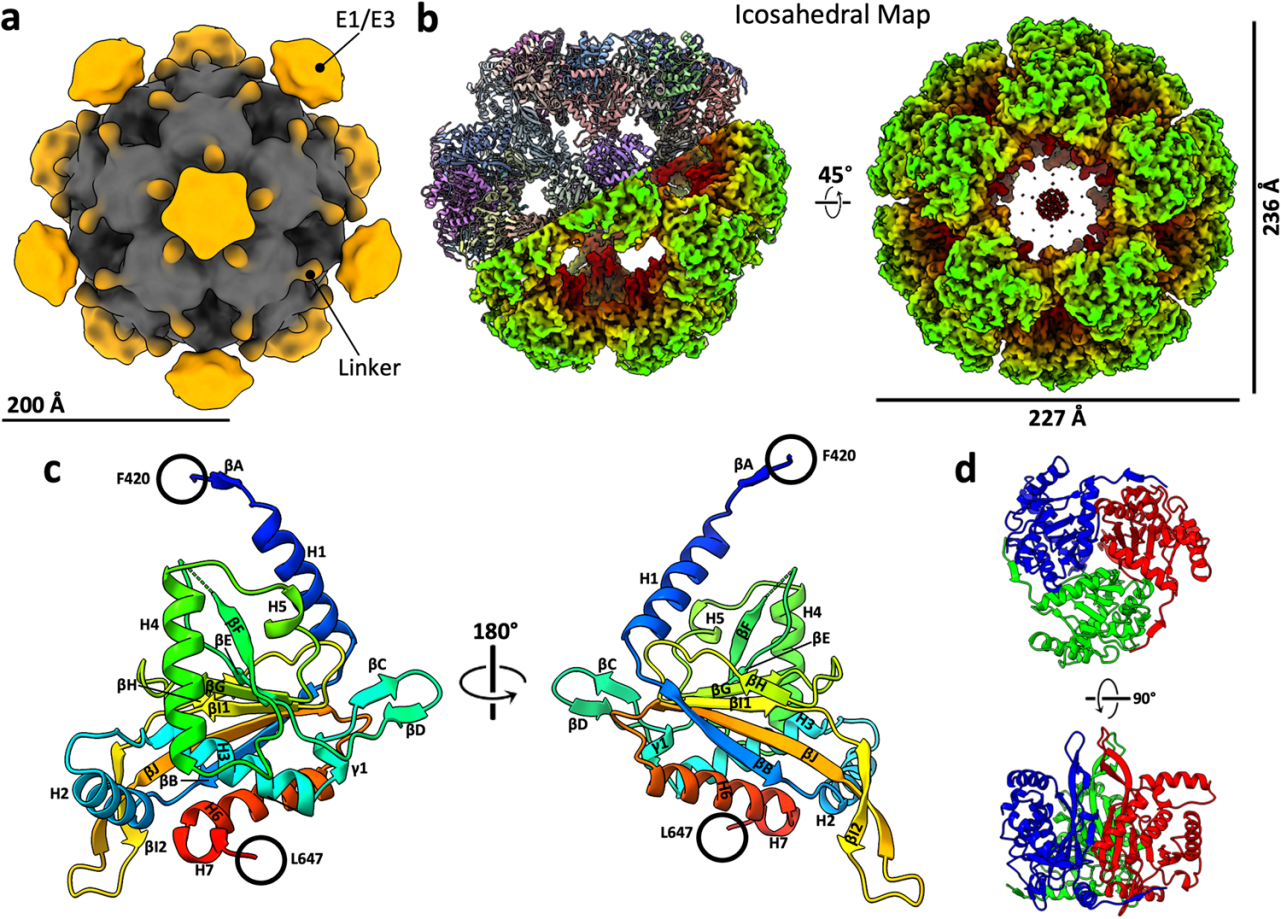
Cryo-EM reconstructions of bovine PDC and atomic structure of native E2p IC domain. **a** 3D reconstruction of the full PDC with icosahedral symmetry shows external densities (orange) around the core density (gray). **b** Two views of the cryo-EM density map of E2p core colored by radius, with our E2p atomic model assembly of a hemisphere shown in the left panel. **c** Rainbow-colored ribbon of E2p IC domain atomic model with secondary structures labeled. Resolvable N-terminal residues (blue) and C-terminus (red) are indicated. **d** Two views of the atomic model of the E2p IC domain trimer, colored by subunits.

To generate samples of intact α-keto acid dehydrogenase complexes, we extracted mitochondrial lysates from bovine (*Bos taurus*) kidney tissue. We then used sucrose gradient fractionation to separate and enrich the protein species present in the mitochondrial lysates (Fig. 1b). Fractions were evaluated by SDS-PAGE, western blot, and mass spectrometry to confirm the presence of PDC and OGDC (Fig. 1b; Supplementary Fig. S1a and Data S1). The mass spectrometry data indicated BCKDC to be present only as a minor species compared to PDC and OGDC. Fractions containing PDC and OGDC were pooled together into a single lysate sample for EM studies. Negative stain 2D class average evaluation of the sample on a single grid showed both icosahedral classes and cubic classes (Supplementary Fig. S2). Negative stain classes also show peripheral densities around the cores, suggesting that E1 or E3 are present and that intact α-keto acid dehydrogenase complexes were recovered. Higher-order assemblies are visible in cryo-EM micrographs (Supplementary Fig. S3a), and smeared densities are present at the periphery of well-resolved cores in the cryo-EM 2D classes (Supplementary Fig. S3b). Notably, additional classes belonging to other unknown protein species were obtained (Supplementary Figs. S2, S3b), thus highlighting the capability of cryo-EM for the study of molecular sociology. Cryo-EM 3D reconstruction yielded cubic and icosahedral cores surrounded by peripheral densities (Figs. 1b, 2a, 3a).

To structurally characterize the putative OGDC and PDC in the pooled lysate, we obtained 3D reconstructions of the cubic and icosahedral cores of the complexes by single-particle cryo-EM at 3.5 Å and 3.8 Å resolution, respectively (Figs. 2b, 3b; Supplementary Fig. S3 and Table S1). We initially identified PDC based on its distinct icosahedral architecture. This is confirmed by atomic model building of the bovine E2p sequence into the icosahedral cryo-EM density map, which showed good agreement between the sequence and the respective side chain densities. FindMySequence results also support the identity assignment of PDC^38^ (*E*-value 4.80e–74, 3.30e–19, and 2.40e–15 for E2p, E2o, and E2b sequences, respectively).

Although PDC is distinct for its icosahedral architecture, both OGDC and BCKDC share cubic architecture, which introduces ambiguity to the identification of cubic α-keto acid dehydrogenase complexes. To confirm whether our cubic complex was OGDC, BCKDC, or a hybrid of both, we created a homology model by mutating a pre-existing human E2o model to the bovine E2o sequence^22^. After fitting and real-space refinement of the model into our cubic cryo-EM density map, the side chains for the E2o model fit the density well (Fig. 2e). Sequence alignment of bovine E2o and E2b reveals primary structure features in E2b that cannot correspond to side chain densities in our map. Additionally, fitting of a pre-existing E2b model into our map showed discrepancies between the modeled side chains and the density^20^. FindMySequence and checkMySequence results also support the identity assignment of OGDC^38,39^ (*E*-value 5.30e–89 and 6.30e–11 for E2o and E2b sequences, respectively). The unambiguous identification of the cubic complex as OGDC is further reflected in the low presence of BCKDC components in the mass spectrometry data.

### Heterogeneous arrangement of peripheral subunits around the native E2 cores

Unmasked 3D reconstructions have external densities suggestive of E1 or E3 around the core (Figs. 2a, 3a). Both OGDC and PDC have a large density over each core face. In PDC, thin densities from the trimer vertices extend toward the density above the pentameric face. OGDC has additional smaller densities along each cubic edge with thin densities of putative linker regions connecting to the E2o trimer vertices. Previous models based on hydrogen/deuterium exchange and cross-linking mass spectrometry studies propose that E1o binds at the edges and E3 binds at the faces^13,22^. The opposite orientation of the small connecting densities between OGDC and PDC suggests different predominant states of activity and a large range of motion for the flexible linker regions. However, enforced symmetry during reconstruction restricts the interpretability of these external densities for the structures and locations of E1 and E3.

To better evaluate the heterogeneous features of the complexes, we performed asymmetric (C_1_) reconstructions of the whole OGDC and PDC followed by a 20 Å low-pass filter and color zone of 16 Å around the fitted E2 core models (Supplementary Fig. S4a, b). External densities corresponding to peripheral subunits are localized at certain faces in both OGDC and PDC (Supplementary Fig. S4a, b), which has been observed in previous C_1_ reconstructions and tomography^32,37,40^. Thin densities suggestive of linkers connect certain external densities to the E2 core.

Notably, in PDC, an unidentified density at the center of the icosahedral reconstruction is also present in the C_1_ reconstruction (Fig. 3b; Supplementary Fig. S4d). This globular density radiates outward from the center of the E2p/E3BP core interior with decreasing contour level, unlike the tetrameric E3BP densities adjacent to the inner E2p core in fungus PDC^16,32,36^. The central density is absent in recombinant E2p and mixed E2p/E3BP cores and endogenous OGDC^30,31^ (Supplementary Fig. S4c), which suggests that the central density is unique to native PDC and does not belong to E2 nor E3BP.

Peripheral subunit densities were further evaluated by symmetry expanded C_1_ sub-particle reconstructions. A 20 Å low-pass filter and color zone of 8 Å around the E2 core model were applied to the sub-particles for greater interpretability of low-resolution features (Supplementary Fig. S4e, f). Although the resolution is too low for unambiguous identification or positioning of E1, E3, LD, or PBSD within the external densities, we utilized the asymmetric sub-particle reconstructions to narrow down their potential localization. Thin densities corresponding to N-terminal linkers originate from both the twofold related intertrimer interface and the N-terminal end of H1 (Supplementary Fig. S4e) in E2o, but the linkers originate from only the H1 site in E2p/E3BP (Supplementary Fig. S4f). A region extending along the N-terminal end of H1 through the preceding linker (residues 224 to 240) has previously been identified as the PSBD in mammalian E2o^13,22^. In native *E. coli* E2p during the resting state, the LD binds to a cleft flanked by the N-terminal end of H1 and the C-terminal end of H4^37^. In both PDC and OGDC, extra densities are located near the H1 sites of E2/E3BP subunits not lining the inner face of interest, and fitting of *E. coli* E2p with bound LDs into the E2o and E2p/E3BP sub-particles supports binding of the LD to these sites^37^. Due to the conflicting role of the H1 site as the PSBD in E2o, the H1 site of E2o may have a dual function as both the LD-binding site and the PSBD, where the binding as either role is mutually exclusive. In PDC, the extra densities at the H1 sites protrude outward and some have thin extensions (Supplementary Fig. S4f, g). These densities have varying prominence and are notably absent at some IC domains (Supplementary Fig. S4g), suggesting a heterogeneous binding of LDs or E1/E3.

The facial density of OGDC localizes around a single trimer vertex and is connected to the E2o IC domains by the linker originating from the H1 sites (Supplementary Fig. S4e). Additional smaller edge densities protrude from the linkers originating from the twofold related interface. In PDC, only a large facial density is observed (Supplementary Fig. S4f). Similar to that of OGDC, the facial density is localized near a trimer vertex and is connected to the IC domain at the H1 site. Linkers from the twofold related interface of E2p/E3BP like those in E2o were not observed. Trajectories of the linker between the IC domain and PSBD from the twofold related interface and from along the threefold axis have been previously observed but only mutually exclusively^16,20–25,30,37,41^. In particular, the N-terminal linker of native *E. coli* E2p in the resting state folds back toward the threefold axis of the trimer after reaching the twofold related interface^37^. Our asymmetric sub-particle reconstructions suggest that the fold of the E2 N-terminal linker is associated with the activity or reaction step of the complex, and both orientations of the linker possibly coexist based on the binding of LD, E1 or E3 (Supplementary Fig. S4e). It remains unknown whether the linker folds back to position the PSBD and LD(s) against its own IC domain or continues past the threefold axis to a neighboring IC domain.

### E2o and E2p utilize distinct interactions to ensure correct self-assembly into E2 trimers and prevent incorrect heterologous binding of different E2 subunits

For E2o, the backbones of residues 220–455 are fully traceable. For E2p, the backbones of residues 420–647 are fully traceable, except for residues 519 and 520, which lack densities. Both E2o and E2p share conserved secondary structure features: six α-helices (H1–H6), a short C-terminal 3_10_-helix (H7), and ten β-strands (βA–βJ) (Figs. 2c, 3c). The secondary structure elements described here are labeled consistently with *E. coli* E2o, bovine E2b, and human E2p^20,30,42^.

The E2 IC domains of OGDC and PDC are arranged as trimers (Figs. 2d, 3d) with 51 interdomain hydrogen bonds and an average buried surface area of 4957 Å^2^ (standard deviation (SD) = 3.47) in E2o and an average of 42.75 (SD = 2.36) interdomain hydrogen bonds and an average buried surface area of 4397 Å^2^ (SD = 22) in E2p. Although E2o and E2p share secondary structure features and overall fold, they have notable differences in certain regions and in the interactions that stabilize their respective trimers (Supplementary Fig. S5a). The N-terminus extends at a less acute angle toward the neighboring IC domain at the clockwise position in E2o than in E2p (Supplementary Fig. S5b). The N-terminus wraps around a turn between βC′ and βD′ (prime symbol is used to denote the neighboring IC domain at the clockwise position from the exterior view), which is longer in E2o than in E2p. The longer βC–βD turn in E2o enables an electrostatic interaction between the negatively charged surface of the turn and the positively charged surfaces of the N-terminus that is not present in E2p (Supplementary Fig. 5d). This positions βA in a β-sheet with βD′ and βC′, and the increased stability allowed for modeling of additional N-terminal residues in E2o. The extended βC–βD turn is also present in human E2o and not E2p, but it is lacking in bacteria (Supplementary Fig. S5c). In addition, E2p possesses a longer interior hairpin (Supplementary Fig. S5a), possibly for engaging in interactions in the larger volume of the icosahedral interior of PDC compared to the cubic interior of OGDC^43^.

**Fig. 4:**
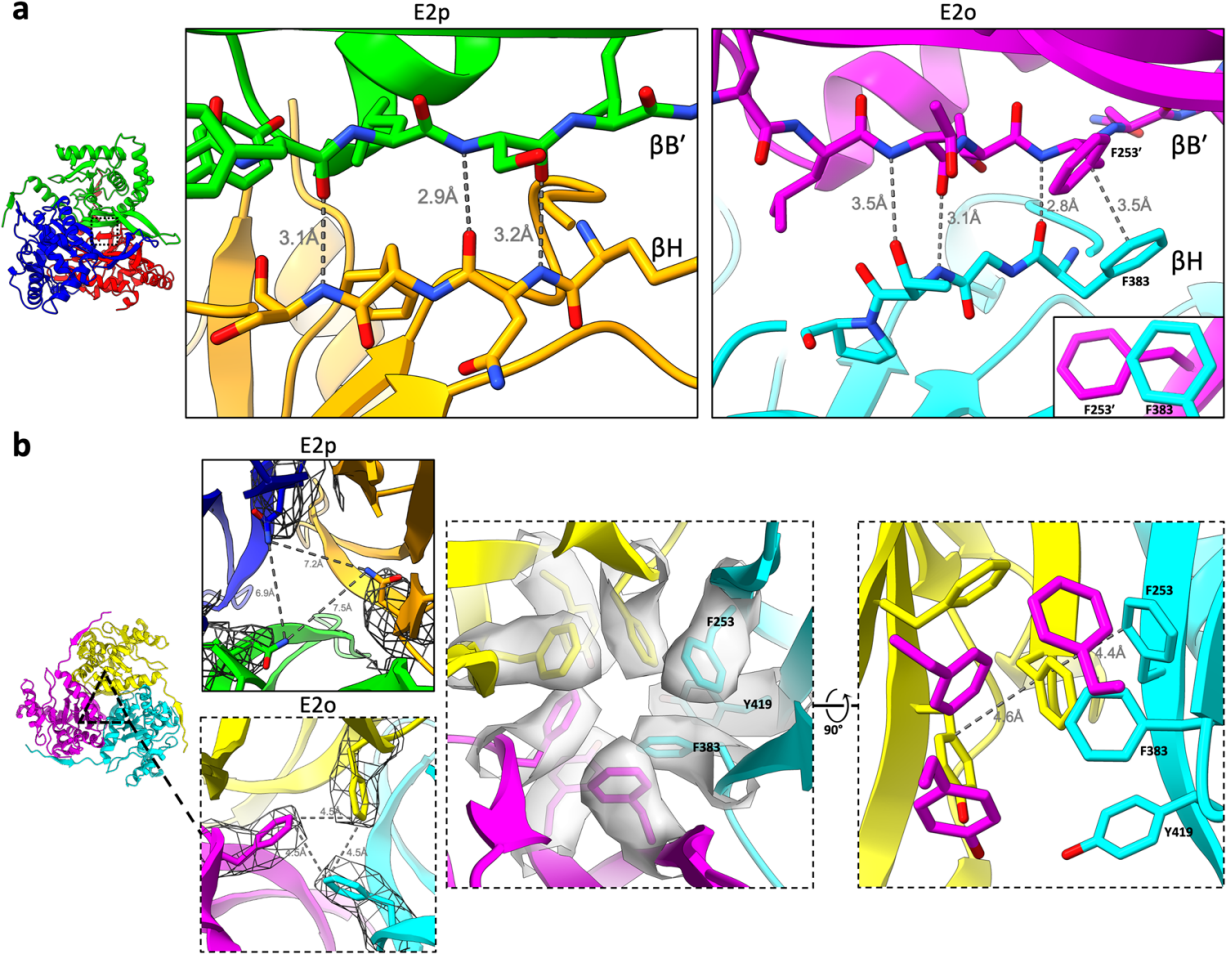
π-stacking interdomain interaction network at the threefold interface. **a** View of the βH–βB′ interface within the interdomain trimer for E2p (left) and E2o (right). βH (orange; cyan) forms three hydrogen bonds with βB′ (green; magenta) in both E2p and E2o. The E2o interface has an additional slipped-parallel π-stacking interaction between Phe383 and Phe253′. Inset shows offset rings from view along the parallel axes. **b** π-stacking interactions present at the threefold axis of E2o trimer but absent in E2p trimer. Expanded views along the threefold axis of E2o show an extended π-electron network formed by F253, F383, and Y419.

Within the IC domain trimer, an E2 subunit binds to its clockwise adjacent neighbor through a short β–β motif between βH and βB′. In E2p, this binding is described by three main chain-to-main chain hydrogen bonds (Fig. 4a). In E2o, this binding is described by two main chain-to-main chain hydrogen bonds, a main chain-to-side chain hydrogen bond, and a slipped-parallel π-stacking interaction^44^ (Phe383–Phe253′). The slipped-parallel π-stacking interaction of E2o occurs near the threefold axis of the trimer and may form a delocalized π-electron network as previously suggested in *Chaetomium thermophilum* E2p between Arg384^36^. By sequence and structure alignment, Phe383 in bovine E2o is the equivalent residue of Arg384 in *C. thermophilum* E2p. Notably, in bovine E2o, the delocalized π-electron network is extended within the threefold axis and encompasses Phe253, Phe383, and Tyr419 (Fig. 4b). This delocalized π-electron network is also present in human E2o but not in bovine E2p at this structurally analogous position because the equivalent bovine E2p residues Asn575 are too distant from each other^22^ (Fig. 4b). Like mismatched puzzle pieces, the different interfaces of E2o and E2p may prevent incorrect assembly due to co-existence of their components within the cell.

### The lipoyl moiety is positioned near a flexible gatekeeper in the catalytic site

**Fig. 5:**
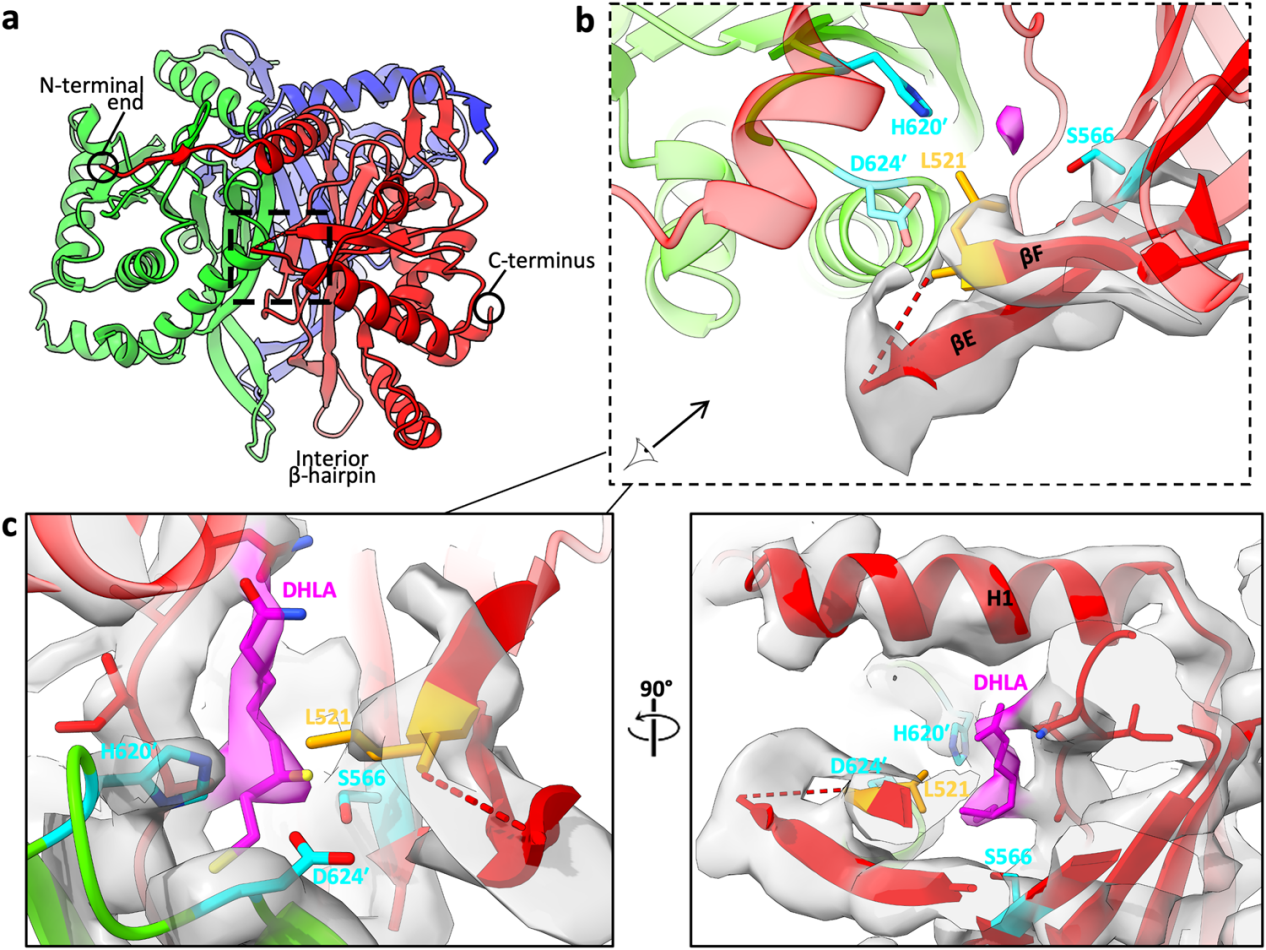
The interdomain active site within the bovine PDC E2 IC domain trimers. **a** Ribbon-model of E2p trimer (colored by subunits) with boxes highlighting the relative locations of the flexible βE–βF turn (black box). **b** Enlarged views of the βE–βF turn near interdomain active site relative to conserved catalytic residues (teal), Ser566 from the red subunit and His620 and Asp624 from the green subunit. Two residues (Ala519 and Gly520) are unmodeled in the turn due to absence of continuous cryo-EM density (semi-transparent gray; for clarity, only those for βE–turn–βF are shown), suggesting flexibility of the residues in the turn. The conserved leucine gatekeeper (orange) is next to these unmodeled residues. A weak density (pink) bordered by catalytic and gatekeeper residues within active site is visible. **c** Cryo-EM density of the same region of **b** shown at a decreased threshold as semi-transparent pink for the lipoyl moiety or gray for the surrounding region. Atomic models for the lipoyl moiety (from PDB ID: 1EAE) and the surrounding area are shown as sticks and ribbon, respectively.

In the cryo-EM density maps of E2p, a region of flexibility has been identified: a β-turn connecting βE and βF near the interdomain active site (Fig. 5a). Due to missing density at the βE–βF β-turn, two residues (Ala519 and Gly520) are unmodelled in our E2p model (Fig. 5b). Although the corresponding density is not missing in E2o, it is still weaker relative to that of the adjacent residues. This missing or weak density has also been observed in previous cryo-EM density maps^16,22,23,25,37^. The density of the adjacent residue (Leu521 in E2p and Leu329 in E2o) is well-defined. This leucine is a conserved gatekeeper residue for the binding of the lipoyl moiety^20,30^. This gatekeeper residue is proximal to the interdomain active site, near the catalytic residues Ser566 and His620′ in E2p, and positions the dihydrolipoamide (DHLA) into the catalytic site during a previously suggested CoA-bound state^20^.

Adjacent to the region of missing density, a putative density for the lipoyl moiety was identified near the catalytic residues Ser566 and His620′ and the gatekeeper residue Leu521 (Fig. 5b). When the contour level is decreased, the density forms a thin extension through the active site channel and accommodates a model of DHLA^45^ (adopted from PDB ID: 1EAE) (Fig. 5c). The E2o cryo-EM density map also contains a putative lipoyl moiety density (Supplementary Fig. S6a). Following the trajectory of these densities, the dihydrolipoyllysine enters the active site from the exterior of the E2 core at an entrance at the end of the LD-binding site cleft between the C-terminal end of H1 and a turn connecting βH and βI1, as previously depicted in *E. coli* E2p in the resting state^37^. Densities of the lipoyl moiety are not present in cryo-EM density maps of native *C. thermophilum* E2p and E2o and *Neurospora crassa* E2p^16,23,36^.

In the E2o density map at low contour level, weak cylindrical densities reminiscent of β-strands from the LD or helices from the PSBD are observed to the left and to the right of the entrance of the cleft. The left densities are located similarly to that of the LD in native *E. coli* E2p in the resting state^37^ (Supplementary Fig. S6b). These additional densities extend along the cleft toward the active site entrance. We attempted to fit LD and PBSD models from NMR (PDB IDs: 1LAC, 1W4H) and AlphaFold prediction^46–49^ (AF-P11179-F1, AF-P11181-F1), but there are mismatches between the cylindrical densities and the LD models when the dihydrolipoyllysine-containing loop is positioned adjacent to the active site channel. These observations combined with structural variation between LD domain models suggest that the β-sheets of the LD may be conformationally variable and undergo conformational and positional changes during catalysis.

To search for E3BP, we expanded the symmetry of the trimer vertex and pentameric face sub-particles and reconstructed them asymmetrically (C_1_) (Supplementary Fig. S7a). Due to low resolution of the C_1_ reconstructions, we are unable to utilize side chain densities to distinguish sequence identity. Sequence alignment between E2p and E3BP and fitting of predicted E3BP structures (PDB ID: 6H60; AF-P22439-F1) show two notable regions of difference in E3BP within the IC domain^31,47,48^: a three-residue shorter linker between H2 and H3 and a three-residue longer interior hairpin between βI2 and βJ (Supplementary Fig. S7b). We searched the classes of the trimer vertex and pentameric C_1_ sub-particles but were unable to identify any differences corresponding to the presence of E3BP (Supplementary Fig. S7c). E3BP is not evenly distributed in the trimers^31^, and current 3D classification methods may place too much weight on the larger features in the conserved fold of E2p and E3BP, which may reduce the quality of asymmetric reconstructions of the interior hairpin. Heterogeneity of E3BP distribution precludes identification and experimental structural determination of E3BP from native mammalian PDC.

### Different knob-and-socket intertrimer interactions prevent heterologous self-assembly of E2 trimers

**Fig. 6:**
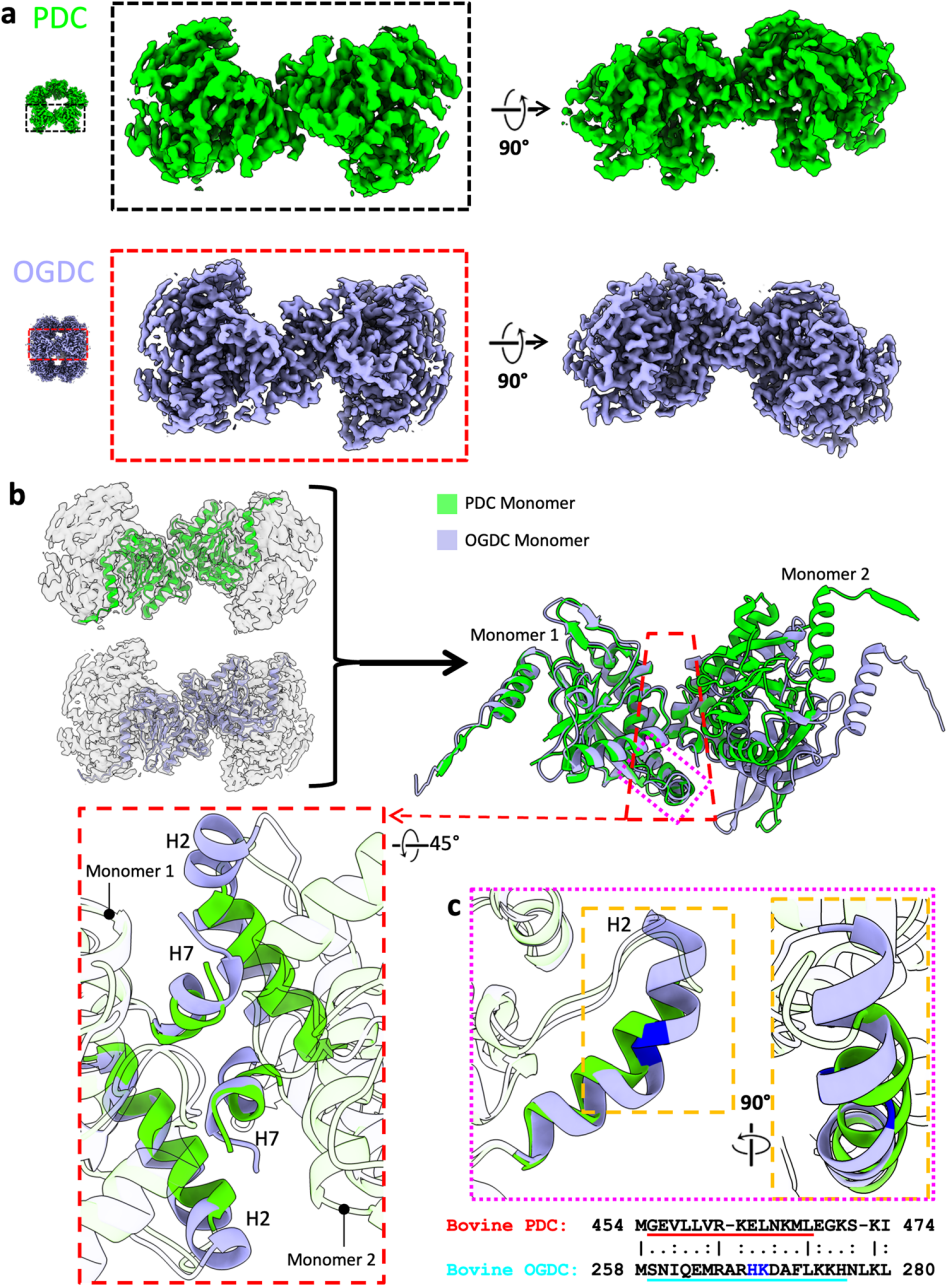
Differences in the E2 intertrimer interactions between PDC and OGDC. **a** Two orthogonal views (perpendicular and parallel to the twofold axis) of interacting E2 trimers from the cryo-EM density maps of PDC (green) and OGDC (blue). **b** Comparison of the intertrimer interface in PDC (green) and OGDC (blue). Interactions involve only two subunits, one from each trimer, as apparent from the left panel where the atomic models (ribbons) of the interacting subunits are superposed in their respective densities. The two interacting subunits shown in **a** are aligned through their left subunit (monomer 1) and shown together to identify differences at the interacting interfaces with boxed regions shown as insets to highlight differences of their corresponding H2 helix. The inset in red box shows the knob-and-socket interaction of H2 and H7 (colored; rest of ribbon transparent for clarity) at twofold interface in E2p and E2o. **c** Superimposition and sequence alignment of H2 in E2p and E2o. The helix–kink–helix (kink highlighted in blue) is present in E2o but absent in E2p. Inset in the orange box: enlarged orthogonal view of the diverging H2 trajectories.

E2o and E2p have near-identical structures, yet they assemble into core scaffolds with different geometry. The cubic and icosahedral geometries are related by principles of quasi-equivalence and Euclidean geometry^24^. Each IC domain trimer vertex of the core is bound to its neighboring trimers through a palindromic twofold related intertrimer interface^22,30^ (Fig. 6a; Supplementary Fig. S8a). The hydrophobic C-terminal 3_10_-helix (H7) of each IC domain binds to a hydrophobic pocket formed by residues of H2*, H7*, and the N-terminal end of H4* (the asterisk is used to denote the partner IC domain across the twofold related interface) on the opposite IC domain in a knob-and-socket interaction (Supplementary Fig. S8b). This binding is further stabilized by electrostatic interactions from oppositely charged surfaces lining the exteriors of H7 and the hydrophobic pocket (Supplementary Fig. S8c). In actinobacteria E2p, where H7 folds back against rather than away from the IC domain, the knob-and-socket interaction cannot occur, so the trimers are unable to assemble into a core^25^.

The symmetries for bovine E2o and E2p are obtained from a different combination of interactions (Supplementary Table S2). The intertrimer interface has an average of 18.7 (SD = 0.49) hydrogen bonds and an average buried surface area of 1102 Å^2^ (SD = 0.46) in E2o and an average of 5.6 (SD = 0.63) hydrogen bonds and an average buried surface area of 825 Å^2^ (SD = 14.18) in E2p. The exterior of the E2o socket is more charged than that of E2p (Supplementary Fig. S8c), which leads to the greater number of hydrogen bonds at the E2o intertrimer interface. In addition, the exterior surface of the socket in E2o and E2p are oppositely charged from one another. Although the residues comprising the hydrophobic sockets between the two species are similar, the shapes of the sockets are different to accommodate likewise different knobs. The E2p knob has one hydrophobic surface that binds a single hydrophobic pocket in the socket. In contrast, the E2o knob has an additional hydrophobic surface on the opposite side of the knob, and the E2o socket contains two pockets to accommodate this difference (Supplementary Fig. S8b). Similar to the purpose of the interdomain interface differences to prevent heterologous assembly of E2 subunits, the oppositely charged exterior surfaces and differently shaped knob-and-socket of the intertrimer interface between E2o and E2p suggest a means of preventing heterologous assembly of E2 trimers during core formation.

To compare the relative positions of trimers at the intertrimer interface between E2o and E2p, the IC domains of the twofold related interface were aligned by superimposition at one trimer vertex (Fig. 6b). The fold of H2 and the position of H7 differ between E2o and E2p. H7 of E2o (Pro448–Leu453) has a length of six residues and has two additional residues (Asp454 and Leu455) appended at the C-terminus. H7 of E2p (Pro642–Leu646) has a length of five residues and one additional residue (Leu647) at the C-terminus. H7 is positioned more closely to H4* in E2p than in E2o, which is enabled in E2p by an additional hydrogen bond between H7 and H4* and fewer hydrogen bonds between H7 and H2* (Supplementary Table S2). In E2o, H2 has a length of 18 residues (Ser259–His276) and is comprised of a helix–kink–helix with the kink starting at residue 268. In E2p, H2 instead has a length of 14 residues (Gly455–Leu468) and is a single, uninterrupted helix (Fig. 6c). The H2 kink in E2o positions the C-terminal end of the helix more closely against H7* for hydrogen bonds between Lys275 and His276 with Asp454* and Asp454*, respectively. The different interactions at the knob-and-socket stabilize E2 IC trimers at angles that enable the Euclidean geometry relationship between a cube and icosahedron.

## Discussion

Advances in cryo-EM and its integration with mass spectrometry analysis have enabled the structural characterization of intact, native complexes^38,50,51^. Endogenous methods have recently been applied to determine structures of PDC in fungi and bacteria^16,23,32,36,37^. Here, we extended these methods to the study of mammalian tissues and determined structures of native bovine OGDC and PDC at sufficient resolutions for model building of their E2 IC domains (Figs. 2, 3). Despite exhaustive classification and refinement of an enormous data set, structures beyond the E2 cores in our asymmetric sub-particle reconstructions were only resolved at low resolution (Supplementary Fig. S4), indicating heterogeneity in the arrangements of the peripheral subunits attached to the core. Nonetheless, analysis of OGDC and PDC from the same source enables direct comparison between the two protein species. Although the E2 IC domains of PDC and OGDC share a nearly identical fold to conduct analogous reactions, OGDC and PDC cores differ in their interdomain (Fig. 4; Supplementary Fig. S5) and intertrimer (Fig. 6; Supplementary Fig. S8 and Table S2) interactions. These nuanced differences suggest a means of modulating self-assembly to mitigate heterologous binding between mismatched E2 species and highlight the divergent evolution of α-keto acid dehydrogenase complexes to meet the requirements of different organisms with varied cellular contents and metabolic environments. In addition, native structures retain biologically relevant features, such as the position of the lipoyl moiety within the E2 active site (Fig. 5; Supplementary Fig. S6a), and present opportunities for discovery in molecular sociology (Supplementary Figs. S4d, S6b) that may be lost in recombinant samples.

Besides the structures reported here, native structures of α-keto acid dehydrogenase complexes from the same endogenous source only became available early this year for the fungus *C. thermophilum*, where both PDC and OGDC were structurally characterized^23^. In contrast to our bovine E2o and E2p structures, the back-folded N-terminal region comprised of βA and H1 is absent in *C. thermophilum* E2p. Interestingly, H1 is still present in *C. thermophilum* E2o from the same size-exclusion chromatography fraction^23^, and is also present in native *N. crassa* and *E. coli* E2p^16,37^. Given that the structured conformation of the N-terminal region is stabilized by electrostatic interactions between βA and H1 with the βC–βD turn (Supplementary Fig. S5d), the back-folded conformation may exist transiently depending on the activity state of the complex.

The unfolded state of H1 has been suggested to be necessary for unhindered insertion of the lipoyl moiety^36^. For *E. coli* E2p in the absence of pyruvate, H1 is folded and binding of the N-terminus extends toward the threefold axis, which was suggested to position and stabilize the E2-LD complex in a resting state^37^. Our sub-particle reconstruction of E2o shows the N-terminal region extending outwards from both the twofold related interface and about the threefold axis simultaneously (Supplementary Fig. S4e), which indicates that multiple conformations of the PSBD-like H1 and preceding linker of E2o are present. The conformation of the linker may correspond to the transient and mutually exclusive binding of either LD to the E2o IC domain or of E1/E3 to the PSBD.

Native structures of PDC and OGDC have been captured in various activity states. While the tertiary structure of E2 remains mostly identical between structures, the conformation of the aspartate (Asp624 in PDC and Asp430 in OGDC of bovine) that is responsible for stabilizing the protonated catalytic histidine within the active site differs. Previous crystallization experiments of *Azotobacter vinelandii* E2p with reduced forms of CoA or dihydrolipoamide yielded structures with the side chain of the aspartate-equivalent (Asn614) facing that of the catalytic histidine while other states of E2p have the aspartate-equivalent rotated away from the histidine^45^. In our E2o and E2p structures, the stabilizing aspartate faces the catalytic histidine (Fig. 5b, c; Supplementary Fig. S6a), which suggests that substrate is bound in the active site. This asparagine is also facing toward the histidine in the native structure of *N. crassa* E2p^16^, but the asparagine is rotated away in the native structures of *C. thermophilum* E2p and E2o and *E.coli* E2p in the resting state^23,36,37^. In recombinant human E2p and E2o, the asparagine is modeled as facing toward the histidine^22,30^. However, the EM density for this asparagine side chain is missing, indicating motility of the side chain in the apo state. There could be a potential mixture of activity or assembly states of OGDC and PDC in different lysate fractions when complexes are sorted by size, and our single-particle cryo-EM analysis of the entire core may have only reconstructed the most stable or populous conformation or a potential hybrid of the active and resting states.

The interdomain interactions between an E2 IC domain and its neighbor are not conserved between E2p and E2o. Bovine and human E2o contain an extensive delocalized π-electron network at the threefold axis (Fig. 4b) that is absent in their E2p counterparts^22,30^. The differences in interdomain interfaces between E2p and E2o within an organism may enable correct self-assembly within a milieu of components in the mitochondrial matrix. *C. thermophilum* E2o may also have a similar delocalized π-electron network, but instead with Met350 at the analogous position of Phe383 in E2o^23^. Met-aromatic motifs can provide additional stabilization compared to purely hydrophobic interactions^52^. Rather than a delocalized π-electron network of aromatic residues, *C. thermophilum* E2p instead possesses a potential stabilizing arginine cluster that is absent in both its E2o counterpart, mammalian E2p, and mesophilic fungus *N. crassa* E2p^16,23,36^. The greater interdomain interactions could contribute toward increased thermal stability needed for thermophiles compared to mesophiles as a result of divergent evolution.

While there are pre-existing structures of α-keto acid dehydrogenase complexes, the diversity of interactions within these complexes between different organisms occludes direct comparison for determinants of assembly. Although *E.coli* E2p and bovine E2b are both cubic like bovine E2o, their interdomain interface interactions differ greatly. The knob of *E. coli* E2p has a semicylindrical hydrophobic surface that complements a narrow, elongated hydrophobic socket^37^. Bovine E2b has a double-sided knob like bovine E2o^20^, but the hydrophobic surfaces are rotated about and have different sizes. Although α-keto acid dehydrogenase complexes can share the same geometry, the interdomain interactions that hold the complex cores together differ between both family members and model organisms. Thus, structural characterization of samples from the same organism is needed to describe distinct interactions.

In mammals, E3BP substitutes for a corresponding E2 in the core scaffold as determined by biochemical analysis instead of being an additional subunit as it is in fungus^16,28,31,32^. Although there is an internal density for our native bovine PDC structure (Supplementary Fig. S4d), the density is centralized and does not resemble the tetrahedral arrangement of fungal E3BP^36^. In addition to the subtle differences between E2p and E3BP in the IC domain (Supplementary Fig. S7b), the linker region between the PSBD and IC domain is twice as long in E3BP compared to that in E2p (Fig. 1a). Due to the short length of the PSBD–IC domain linker in E2p, E1p is expected to be close to the core. Likewise, the longer linker in E3BP enables binding of E3 further away from the core, possibly to better accommodate the larger size of E3 compared to E1p. Because of the heterogeneous arrangement of E3BP and peripheral E1 and E3 subunits, PDC and other α-keto acid dehydrogenase complexes may need to be studied at the individual level. Tomography could identify peripheral subunits and avoid issues of classification for E3BP if sufficient resolution could be reached in the future.

Advancements in cryo-EM have enabled atomic modeling of native α-keto acid dehydrogenase complexes and other endogenous complexes. Looking forward, faster direct electron detectors and improved cryo-EM grid preparation methods will improve acquisition of particles for low-population species for unambiguous identification and atomic modeling. Heterogeneous reconstruction could enable visualization of distinct protein conformations^53,54^. Machine-learning-based methods in particle picking^55^, structure prediction^47^, and identification can resolve unknown identities in a society of proteins^38^. Structural determination of native geometrically variable complexes from similar folds, exemplified by the cubic and icosahedral complexes presented here, should not only provide new biological insight but also inform protein engineering applications by providing an opportunity for comparison within the same environment to identify the determinants of protein architecture.

## Materials and methods

### Preparation of bovine mitochondria

Bovine mitochondria were prepared from *B. taurus* kidneys as described with modifications^56^. 6 kg of bovine kidneys were collected and chilled on ice immediately after slaughter. Cortical tissues were cut into small slices (0.5–1 cm) and soaked in 4 L water for 1 h and then washed with 2 L kidney buffer (20 mM potassium phosphate, pH 7.6, 250 mM sucrose, 1 mM EDTA). The slices were passed through an electric meat grinder, and the ground meat was suspended in kidney buffer with 10 mM 2-mercaptoethanol and diluted to a final volume of 12 L. The suspension was homogenized using an Ultra-Turrax (IKA) homogenizer (40 s), filtered through 8 layers of cheesecloth accompanied with a sieve, and diluted to a total volume of 18 L using kidney buffer. The resulting supernatant was centrifuged at 2000–3000× *g* for 10 min. The supernatant was decanted and further centrifuged at 5600× *g* for 25 min. The lysosome-enriched fluffy layer was carefully removed from the mitochondrial pellet. The pellets were resuspended in a total of 8 L kidney buffer and filtered again through 8 layers of cheesecloth. The suspension was homogenized using an Ultra-Turrax homogenizer (30 s), diluted to 12 L with kidney buffer, and centrifuged at 5600× *g* for 25 min. The pellets were resuspended with kidney buffer with 0.2 mM PMSF, 0.25 μg/mL aprotinin, 0.14 μg/mL pepstatin, and 1 μg/mL leupeptin in a total volume of 4 L. The suspension was treated with 0.01% digitonin for 15 min to remove outer mitochondrial membranes and diluted to 12 L using kidney buffer. The resulting mitoplasts were concentrated by centrifugation at ~25,600× *g* for 20 min followed by removal of excess kidney buffer. The pellet of the final step was flash frozen in liquid nitrogen and stored at −80 °C.

### Isolation of bovine PDC and OGDC complexes

100 g of frozen mitoplasts were thawed in 150 mL lysis buffer (20 mM potassium phosphate, pH 7.6, 50 mM NaCl, 0.02 mM thiamin pyrophosphate, 20 mM MgCl_2_, 2 mM EGTA, protease inhibitors (2 mM PMSF, 2.5 μg/mL aprotinin, 1.4 μg/mL pepstatin, and 10 μg/mL leupeptin)). The sample was diluted to 337.5 mL with lysis buffer. 37.5 mL Triton X-100 buffer (lysis buffer components in addition with 16% (v/v) Triton X-100) was then added, and the sample was stirred for 15 min at 4 °C. The suspension was centrifuged (SLA-3000, 13,000 rpm, 20 min, 4 °C), and the supernatant was PEG precipitated in 5% (w/v) PEG 10,000 for 15 min. The precipitate was collected by centrifugation (2500× *g*, 7 min, 4 °C) and resuspended in 35 mL lysis buffer. The suspension was homogenized using a Dounce homogenizer and centrifuged (SW28 Ti rotor, 28,000 rpm, 17 min, 4 °C). The resulting supernatant was loaded onto 50% (w/v) sucrose cushions (15 mL) and centrifuged (SW41 rotor, 40,000 rpm, 24 h, 4 °C). Pellets were each dissolved in 0.5 mL sample buffer (50 mM MOPS-KOH buffer, pH 7.0, 0.02 mM thiamin pyrophosphate, 10 mM MgCl_2_, 0.1 mM EGTA, protease inhibitors, 1 mM DTT) by shaking at 230 rpm for 1 h and clarified by centrifugation (SLA-3000, 16,000 rpm, 20 min, 4 °C). The sample was loaded onto 10%–40% (w/v) sucrose gradients (1 mL per gradient) and centrifuged (SW41 Ti, 26,000 rpm, 12 h, 4 °C). The gradients were fractionated and assessed by SDS-PAGE and western blot using a PDC complex antibody cocktail (Abcam) that contains four different mAbs reacting specifically with E1α, E1β, and E2/E3BP. The fractions containing PDC and OGDC complexes were pooled, dialyzed to final buffer (50 mM MOPS-KOH buffer, pH 7.0, 0.02 mM thiamin pyrophosphate, 1 mM MgCl_2_, 1 mM NAD^+^, 0.1 mM EGTA, protease inhibitors, 1 mM DTT), and concentrated for cryo-EM analysis. Liquid chromatography-tandem mass spectrometry was performed on the sucrose gradient fractions. Gradient fractions corresponding to OGDC were subjected to gel filtration on a Superose 6 column (GE Healthcare) to remove ferritin.

### Cryo-EM sample preparation and image acquisition

Cryo-EM grids were prepared by using an FEI Mark IV Vitrobot (Thermo Fisher Scientific). 3 μL of sample was applied onto a glow-discharged lacey carbon copper grid with a thin, continuous carbon film (Ted Pella). After waiting for 30 s, the grid was blotted (8 °C, 100% humidity, 10 s blotting time, 1 blotting force) and plunge-frozen in liquid ethane. Grids were loaded into a Titan Krios (Thermo Fisher Scientific) equipped with a Gatan Imaging Filter (GIF) Quantum LS and a Gatan K2 Summit direct electron detector. Movies were recorded with SerialEM at super-resolution mode^57^. The nominal magnification was 105,000×, corresponding to a calibrated pixel size of 0.68 Å at the specimen level. The total exposure time for each movie was set to 8 s and fractionated equally into 40 frames, leading to a total dosage of ~45 electrons/Å^2^. Defocus was set from –1.8 to –2.6 μm.

### Image processing

The movies were motion-corrected with MotionCor2^58^. 6959 good images were selected from a total of 9029 by manual screening. After defocus determination by CTFFIND4^59^, particles were automatically picked using Gautomatch (https://www2.mrc-lmb.cam.ac.uk/research/locally-developed-software/zhang-software/). 33,138 particles for PDC complex and 41,165 particles for OGDC complex were obtained by 2D and 3D classification in RELION-3^60^. FSC calculations were performed in RELION-3.

For PDC complex, all particles from the previous step were recentered and extracted with a box size of 576 pixels. These particles were refined with icosahedral symmetry, resulting in a map with a resolution of 3.8 Å. To get better density for the peripheral E1 and E3 subunits, a structure-guided sub-particle strategy was used. First, the STAR file from refinement was expanded with icosahedral symmetry, leading to a total of 1,988,280 particles. Second, the center of the expanded particles was shifted to one trimer or pentameric face. Sub-particles were extracted with a box size sufficient for accommodating E1 and E3. Finally, 3D classifications with a local mask focused on either the internal or external region were performed. No classes with identifiable features were obtained for the internal region. For the external region, one class with 13.4% of all particles best displays a connecting density between E2p and the putative E1/E3/LD/PSBD. The particles in this class were subjected to further classifications with different parameters (skip-align classification and local search classification), but they did not yield improved density maps for E1/E3/LD/PSBD.

For OGDC complex, all particles from the previous step were recentered and extracted with a box size of 384 pixels. These particles were refined with octahedral symmetry and a mask around the E2 core, resulting in a map with a resolution of 3.5 Å. To get better density for the peripheral E1 and E3 subunits, a structure-guided sub-particle strategy was used. First, the STAR file from refinement was expanded with octahedral symmetry, leading to a total of 987,960 particles. Second, the center of the expanded particles was shifted to one face of the cubic core, and particles were extracted in RELION with a box size of 208. Finally, 3D classification with a local mask of the external area was performed. One class with 12.7% of all particles showed the best connections between E2 and the exterior E1/E3. The particles in this class were subjected to further classifications with different parameters (skip-align classification and local search classification). However, no maps with better density of exterior E1/E3/LD were obtained.

### Atomic modeling

For model building, human E2o (PDB ID: 6H05) and human E2p (PDB ID: 6CT0) were used as templates for bovine E2o and E2p IC domains, respectively^22,30^. Each monomeric subunit model was fitted into the respective cryo-EM density map of the core scaffold using ChimeraX^61^, mutated and manually refined in Coot^62^, and real-space refined in Phenix^63^. The monomer model was then duplicated to the appropriate stoichiometry and fitted into the respective map and refined iteratively using ISOLDE and Phenix^64^. FindMySequence and checkMySequence were later used to confirm the identity of OGDC^38,39^. Electrostatic potential maps were calculated in ChimeraX. Sequence alignments were performed with Clustal Omega and EMBOSS Needle on the EMBL-EBI server^65–67^. Structure predictions were obtained from AlphaFold using ColabFold or the EBI database^47,48,68^.

## Supplementary information

Supplementary Information

Supplementary Data S1

## Data Availability

Mass spectrometry data and source gel/blot images are provided with this paper. Cryo-EM density maps of the cubic bovine OGDC core and icosahedral bovine PDC core have been deposited in the Electron Microscopy Data Bank under accession numbers EMD-26649 and EMD-26650, respectively. The coordinates of the E2o IC domain and bovine E2p IC domain have been deposited in the Protein Data Bank under accession numbers 7UOL and 7UOM, respectively.
