# Supplementary material for: Structures and comparison of endogenous 2-oxoglutarate and pyruvate dehydrogenase complexes from bovine kidney
Source: Cell Discov. 2022 Nov 22;8:126. doi: 10.1038/s41421-022-00487-y (PMC9681731; doi:10.1038/s41421-022-00487-y)
Supplement: Supplementary file 1 — Supplementary Information [file 41421_2022_487_MOESM1_ESM.pdf]

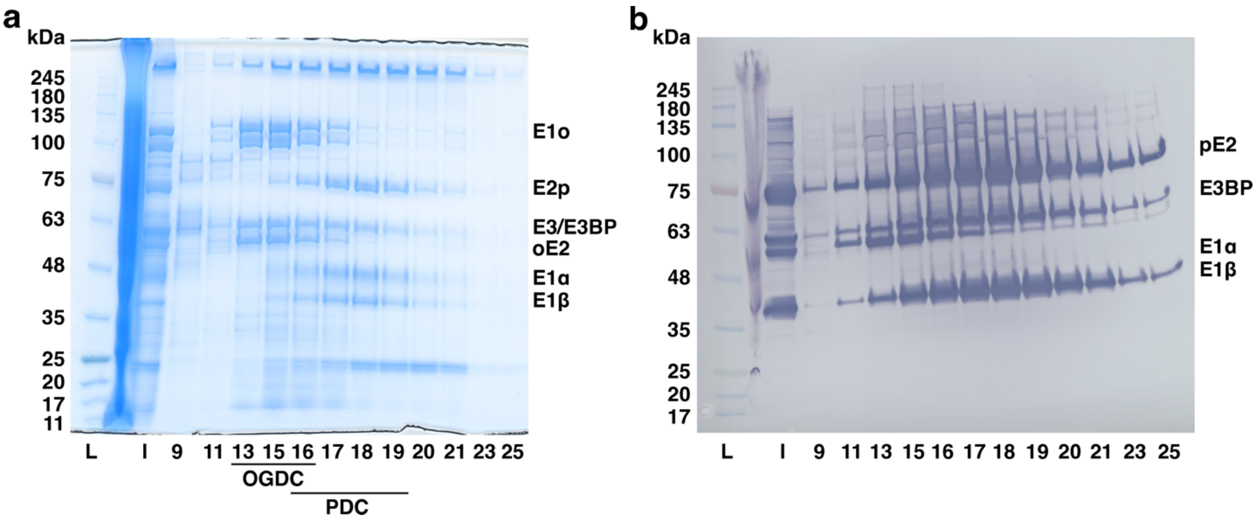

Supplementary Fig. S1. Raw images of gels and blots. (a) SDS-PAGE. **(b)** Immunoblot with PDC antibody cocktail. Numbers below lanes indicate sucrose gradient fraction. Underline indicates the predominant fractions containing OGDC or PDC. Lane I shows input for sucrose gradient.

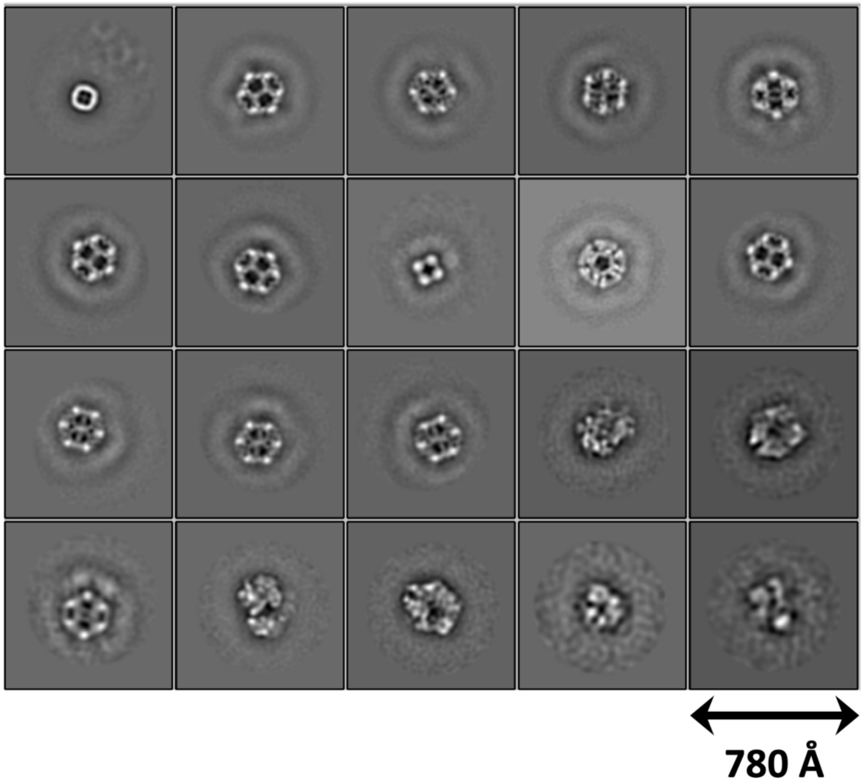

Supplementary Fig. S2. Negative stain 2D class averages. Negative stain 2D class averages show cubic and icosahedral cores with peripheral density. Additional classes of unknown protein species were also observed.

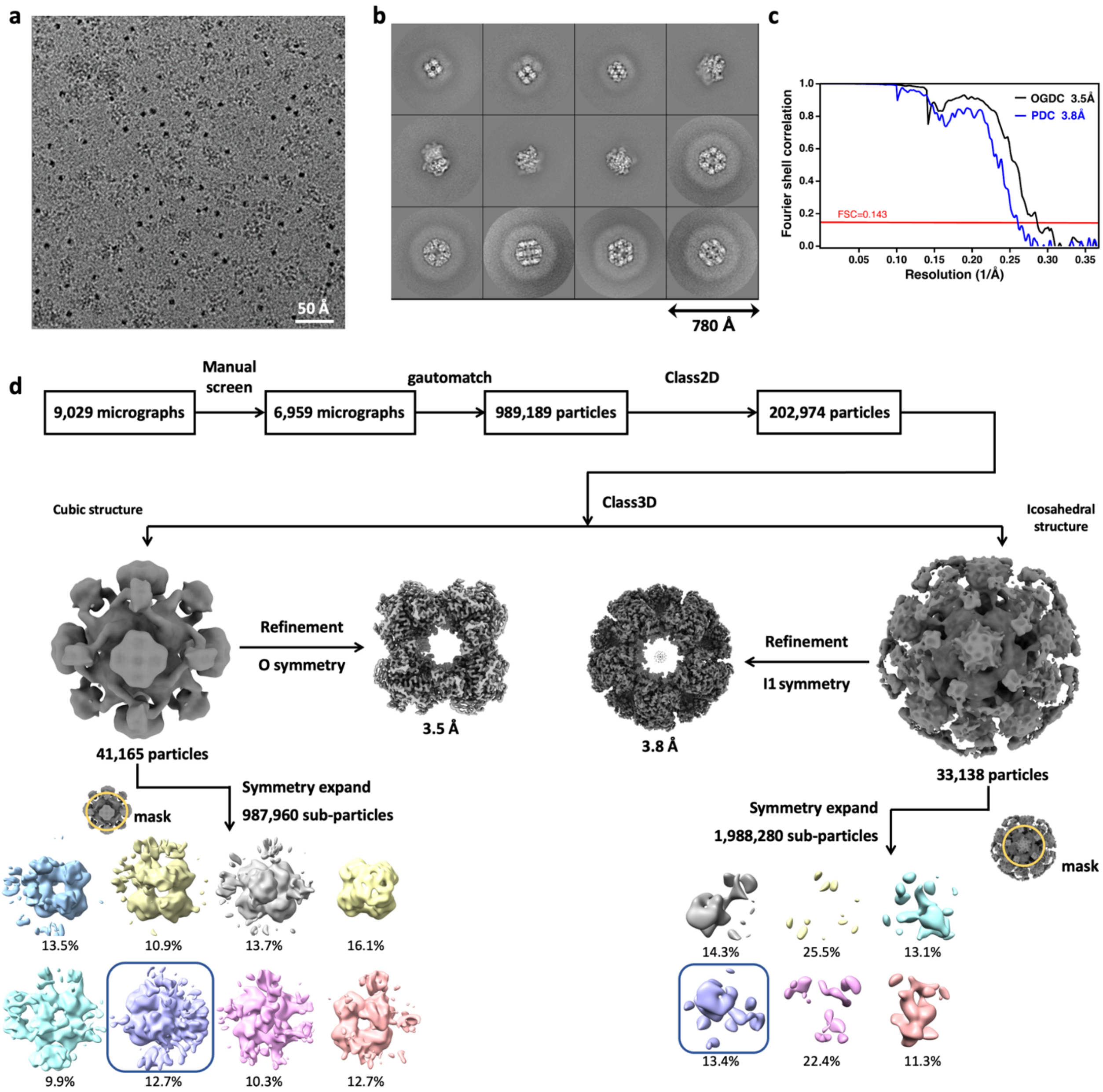

Supplementary Fig. S3. CryoEM data processing workflow. (a) Representative cryoEM micrograph of lysate containing PDC and OGDC. **(b)** CryoEM 2D class averages depicting icosahedral and cubic cores with smeared peripheral density. Classes of additional unidentified protein were also obtained. **(c)** FSC of E2p and E2o core. **(d)** Data processing workflow for reconstruction of E2o and E2p cores and sub-particles.

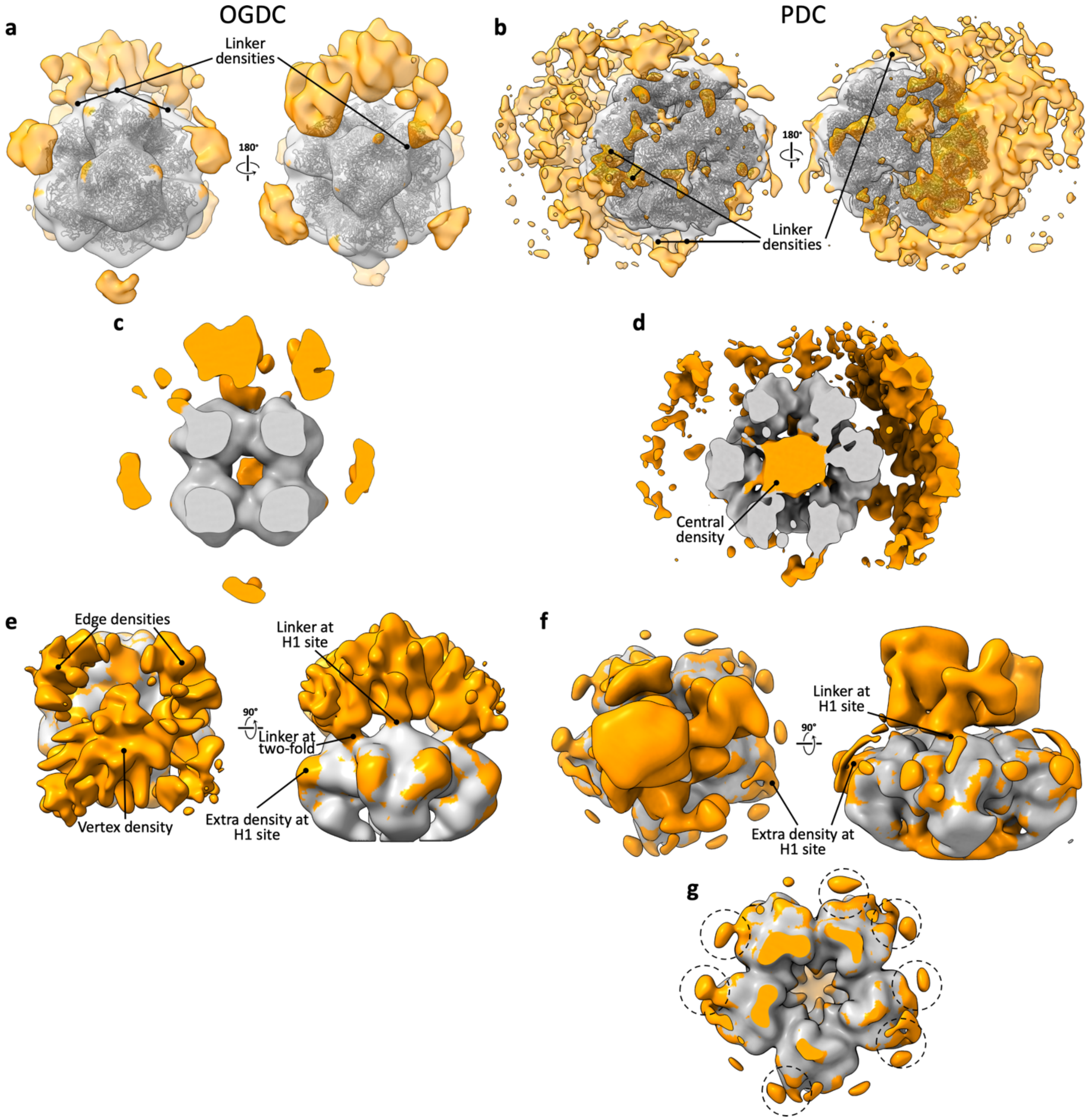

Supplementary Fig. S4. Asymmetric (C_1) whole complex and sub-particle reconstructions of OGDC and PDC. (a) Two views of C_1 reconstruction of whole OGDC low-pass filtered to 20 Å and with density external to fitted E2o model (grey) in orange. **(b)** Two views of C_1 reconstruction of whole PDC low-pass filtered to 20 Å and with density external to fitted E2p model (grey) in orange. **(c)** Sliced view through center of C_1 reconstruction of whole OGDC. **(d)** Sliced view through center of C_1 reconstruction of whole PDC. Central density within E2p/E3BP core is indicated. **(e)** Top (left) and side (right) views of sub-particle reconstruction with C_1 symmetry of cubic face of OGDC. **(f)** Top (left) and side (right) views of sub-particle reconstruction with C_1 symmetry of pentameric face of PDC. **(g)** Low-pass filtered (20 Å) OGDC sub-particle map with density external to fitted E2o model in orange. **(h)** Low-pass filtered (20 Å) PDC sub-particle map with density external to fitted E2p model in orange. **(i)** Sliced view of PDC sub-particle at H1 sites. Additional densities at H1 sites are circled.

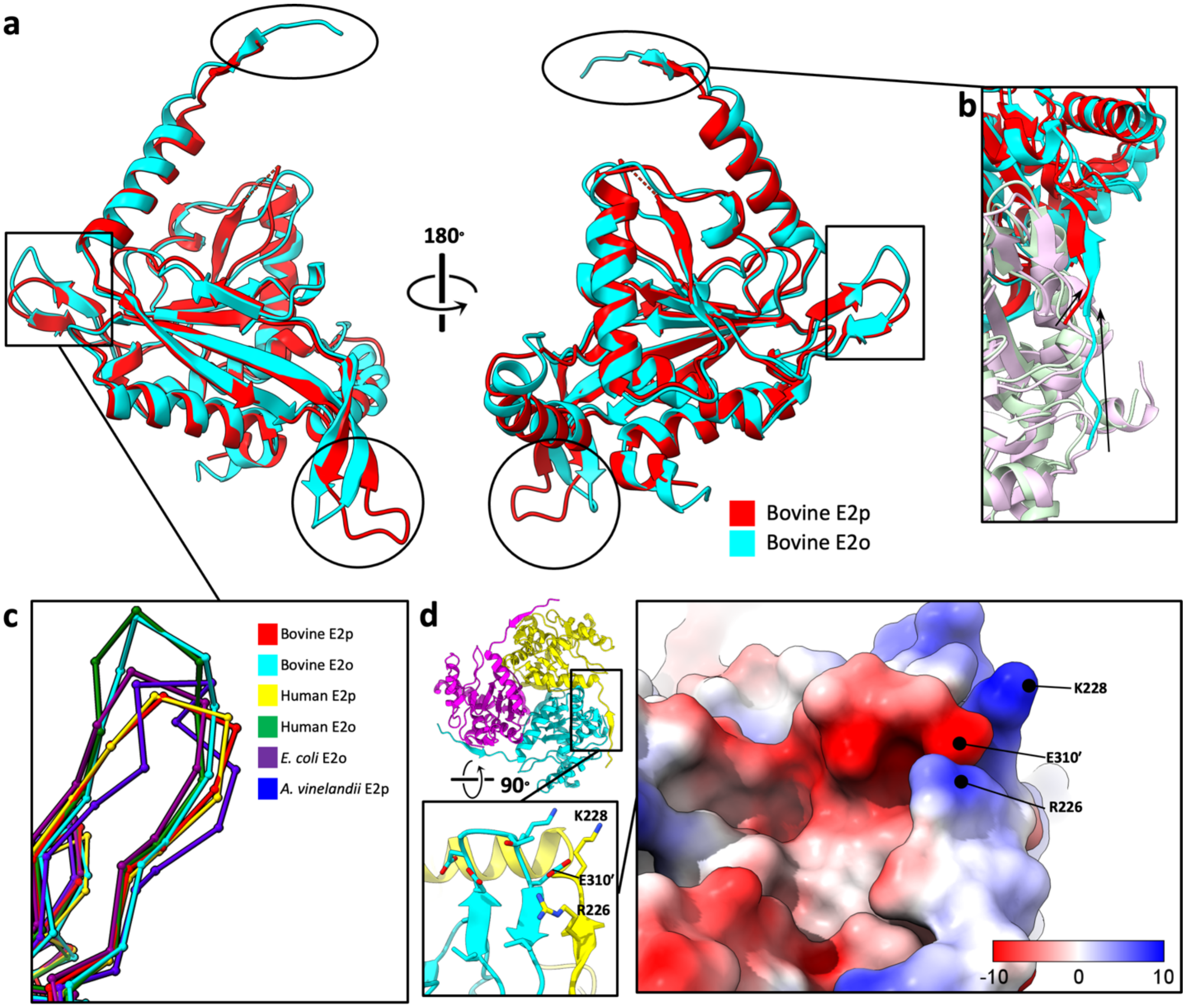

Supplementary Fig. S5. Structure comparison between PDC and OGDC E2 IC domains. **(a)** Superimposition of E2p and E2o IC domains with the N-terminal linker, βC-βD turn, and interior βI2-βJ hairpin indicated by an oval, box, and circle, respectively. E2p has a longer interior hairpin than that of E2o (circle). N-terminal tail diverges at different angles between E2o and E2p (oval). E2o has a more extended βC-βD turn than that of E2p (box). **(b)** Rotated view showing N-terminal tails of E2o and E2p relative to their clockwise neighbor. Arrows indicate trajectories of N-terminal tails towards βA. **(c)** Comparison of βC-βD turn from E2o and E2p of various organisms. **(d)** Location of N-terminal linker and βC-βD turn contact in the E2o trimer. Lower inset shows residue interactions between R226 and K228 of N-terminal linker from the yellow subunit with E310′ of βC-βD turn from the cyan subunit. Right inset: electrostatic surface potential of the same area as that in the lower inset. The positively charged N-terminal linker (primarily blue) associates with the negatively charged βC-βD turn (primarily red).

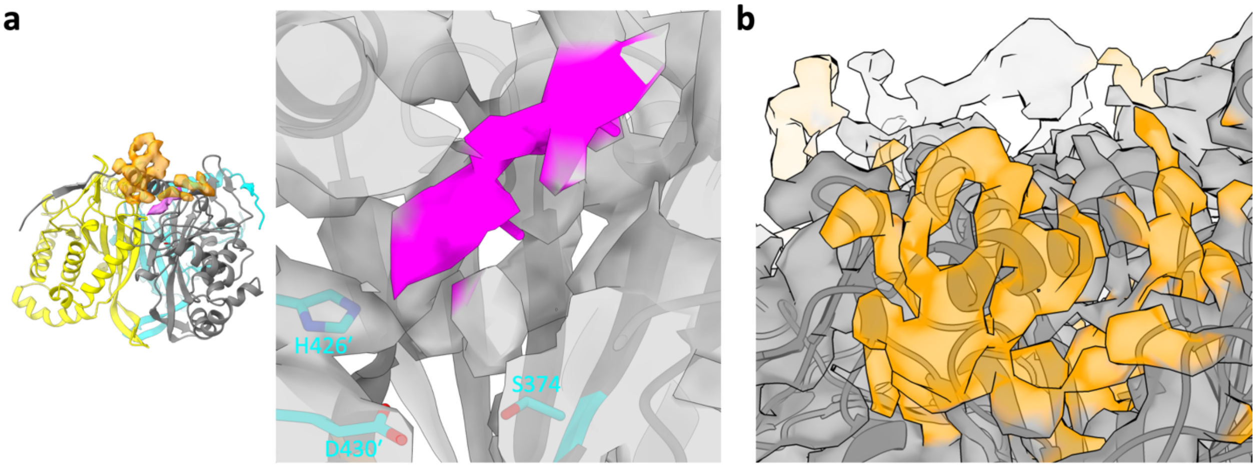

Supplementary Fig. S6. Lipoyl moiety and tentative lipoyl domain density in native E2o. (a) (left) Additional cryoEM density at active site (magenta) and near cleft between H1 and turn connecting βH and βI1 (orange). (right) Enlarged view of weak, thin density within active site channel of E2o corresponding to putative lipoyl moiety (magenta). Catalytic residues (teal) indicated for reference. **(b)** Extra, cylindrical density (orange) located near the previously proposed LD binding site.

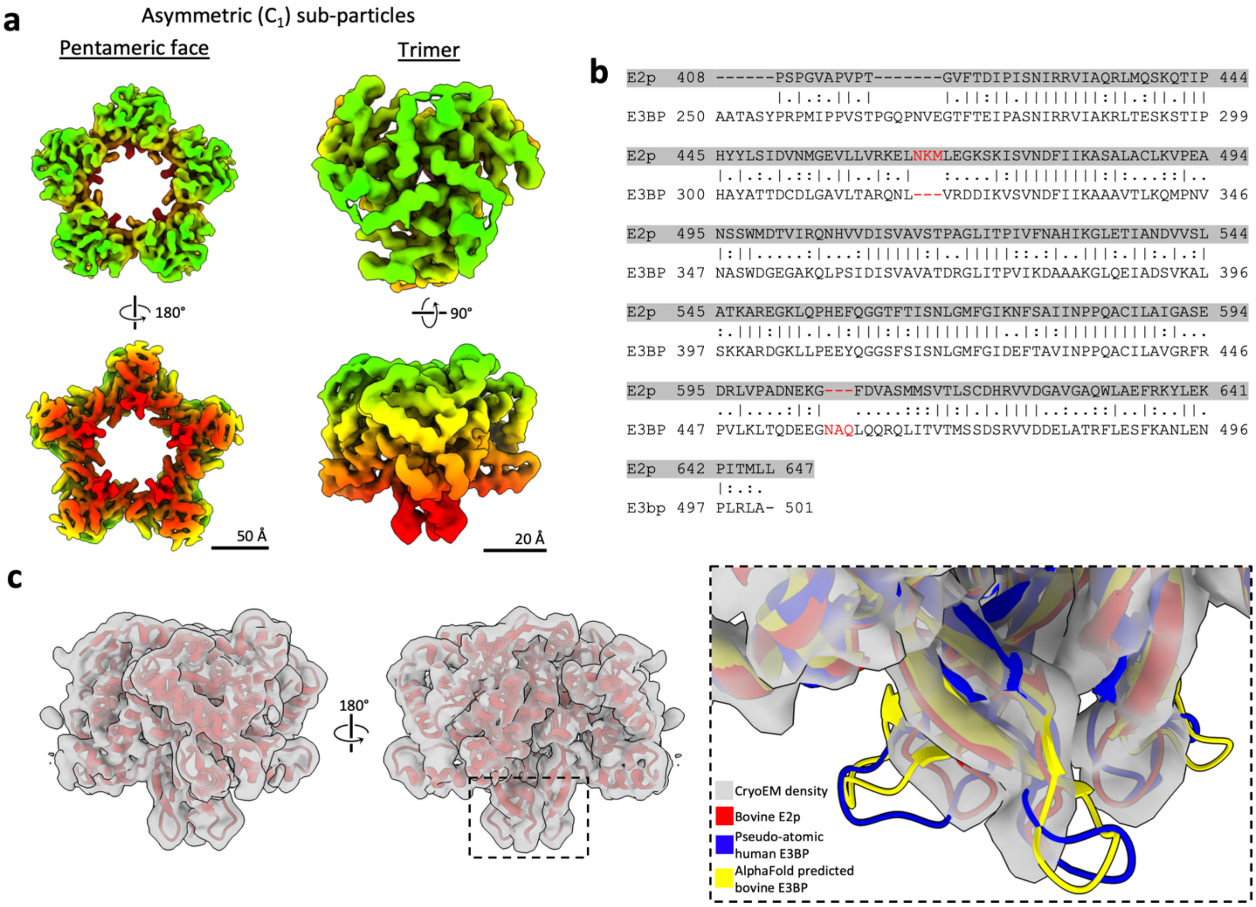

Supplementary Fig. S7. Search for E3BP in asymmetric sub-particle reconstructions. (a) Representative pentameric face (left) and trimer (right) sub-particle reconstructions with C_1 symmetry. **(b)** Sequence alignment between bovine E2p and E3BP. Three-residue differences are indicated in red. **(c)** Atomic model of E2p IC domain fit into trimer sub-particle reconstruction with C_1 symmetry. The βI2-βJ interior hairpin in E3BP is extended compared to that of E2p and is a key feature for distinguishing the two components. All cryoEM densities of interior hairpins from each trimer class appear identical and fit E2p closely. Enlarged view of the boxed area depicts atomic models fit into cryoEM density of interior hairpin. The extended hairpins of predicted human and bovine E3BP models (PDB ID: 6H60; AF-P22439-F1) are not accommodated by the cryoEM density.

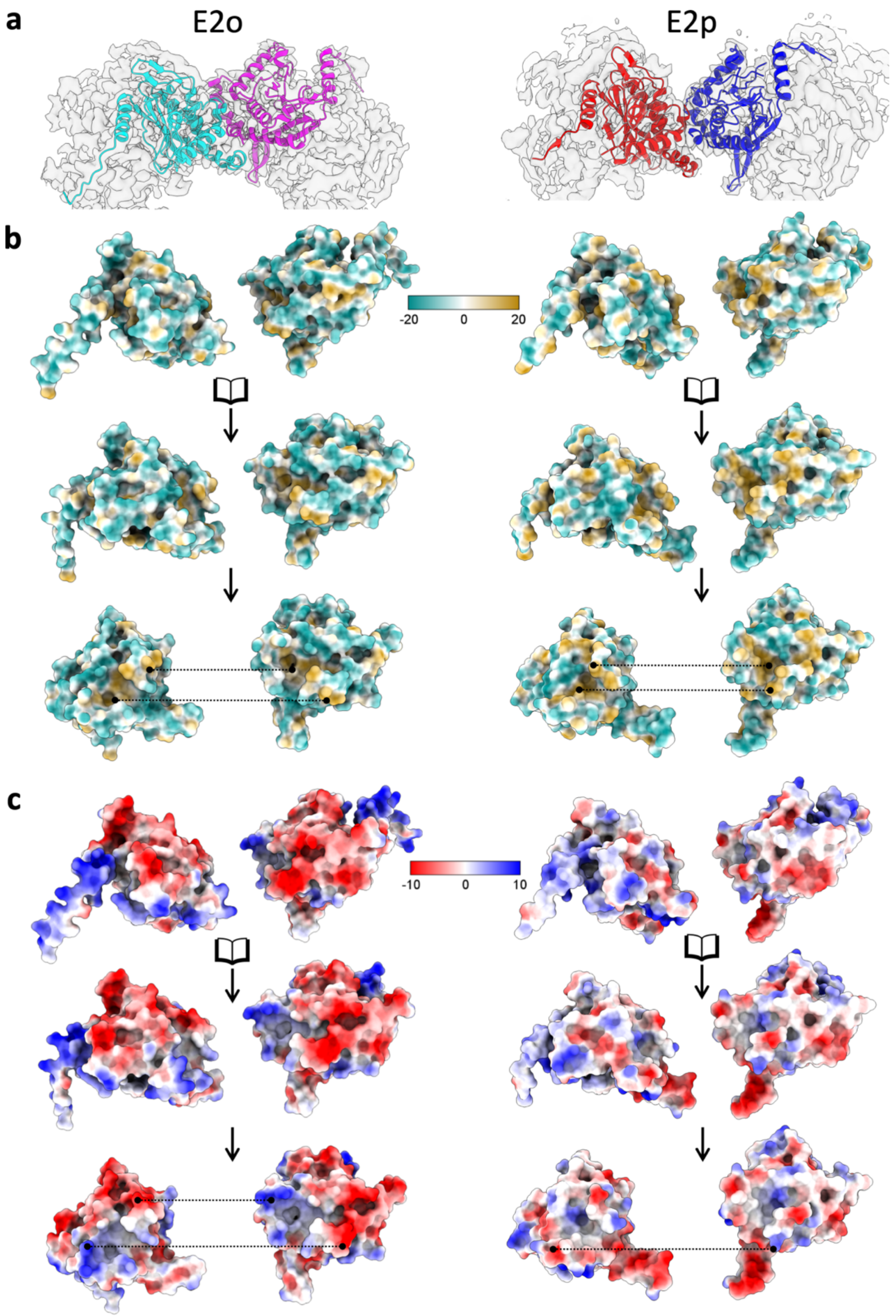

Supplementary Fig. S8. Electrostatic surface potential and hydrophobicity of E2o and E2p two-fold related intertrimer interface. (a) Two E2o (left) and E2p (right) IC domains at two-fold related intertrimer interface superposed in their respective density (transparent grey) and colored by chain. **(b**) Open-book view of hydrophobicity at intertrimer interface. A hydrophobic knob on each subunit binds to a complementary hydrophobic pocket on the partner subunit across the interface. Dotted lines indicate contacting surfaces. **(c)** Open-book view of electrostatic surface potential at intertrimer interface. Oppositely charged surfaces on the exterior mediate electrostatic interactions between the two subunits at the intertrimer interface.

Supplementary Table S1. CryoEM data collection, refinement, and validation statistics

	#1 OGDC (EMDB-26649) (PDB 7UOL)	#2 PDC (EMDB-26650) (PDB 7UOM)
**Data collection and processing**		
Magnification	105,000 ×	105,000 ×
Voltage (kV)	300	300
Electron exposure ($e^{-}/Å^2$)	45	45
Defocus range (μm)	-1.8 to -2.6	-1.8 to -2.6
Pixel size (Å)	1.36	1.36
Symmetry imposed	O	I1
Initial particle images (no.)	989,189	989,189
Final particle images (no.)	41,165	33,138
Map resolution (Å)	3.5	3.8
FSC threshold	0.143	0.143
Map sharpening B factor ($Å^2$)	-152	-150
**Refinement**		
Model-to-map fit, mask CC	0.88	0.87
Model composition		
Non-hydrogen atoms	44496	104640
Protein residues	5664	13560
Ligands	0	0
Mean Isotropic *B* factors ($Å^2$)		
Protein	46.08	78.36
Ligand	N/A	N/A
R.m.s. deviations		
Bond lengths (Å)	0.004	0.004
Bond angles (°)	0.944	0.940
Validation		
MolProbity score	1.13	1.39
Clashscore	3.05	7.12
Rotamer outliers (%)	0.5	0.09
Ramachandran plot		
Favored (%)	97.86	98.54
Allowed (%)	2.14	1.46
Outliers (%)	0.00	0.00

Supplementary Table S2. Interactions at the intertrimer interface

Interactions	Cubic E2o core	Icosahedral E2p core
Hydrogen bonds (donor-acceptor)		
H2 and H7 interaction		
	Arg267-Asp447	(Lys641-Glu463)
	His268-Asp447	
	Lys275-Asp454	
	(Lys275-Asp455)	
	His276-Asp454	
	Arg449-Asn260	
	Arg449-Glu263 [×3]	
H4 and H7 interaction		
	Tyr342-Leu452	Leu534-Leu646
		Glu535-Leu647
Hydrophobic residues composing knob		
	Leu452	Ile643
	Leu453	Leu646
	Leu455	
Hydrophobic residues lining socket		
	Met264	Val460
	Phe272	Leu464
	Leu280	Phe479
	Tyr342	Leu534
	Pro448	Pro642
	Leu451	Met645

Duplicate two-fold interactions not listed. Parentheses indicate interface-variable interaction. Equivalent interactions indicated by matching colors. Arg449-Glu263 share three hydrogen bonds.
